# Cold Kit Labeling: The Future of ^68^Ga Radiopharmaceuticals?

**DOI:** 10.3389/fmed.2022.812050

**Published:** 2022-02-10

**Authors:** Nicolas Lepareur

**Affiliations:** ^1^Comprehensive Cancer Center Eugène Marquis, Rennes, France; ^2^Univ Rennes, Inrae, Inserm, Institut NUMECAN (Nutrition, Métabolismes et Cancer), UMR_A 1341, UMR_S 1241, Rennes, France

**Keywords:** cold kit, gallium-68, molecular imaging, positron emission tomography (PET), radiolabeling, radiopharmaceuticals

## Abstract

Over the last couple of decades, gallium-68 (^68^Ga) has gained a formidable interest for PET molecular imaging of various conditions, from cancer to infection, through cardiac pathologies or neuropathies. It has gained routine use, with successful radiopharmaceuticals such as somatostatin analogs ([^68^Ga]Ga-DOTATOC and [^68^Ga]GaDOTATATE) for neuroendocrine tumors, and PSMA ligands for prostate cancer. It represents a major clinical impact, particularly in the context of theranostics, coupled with their ^177^Lu-labeled counterparts. Beside those, a bunch of new ^68^Ga-labeled molecules are in the preclinical and clinical pipelines, with some of them showing great promise for patient care. Increasing clinical demand and regulatory issues have led to the development of automated procedures for the production of ^68^Ga radiopharmaceuticals. However, the widespread use of these radiopharmaceuticals may rely on simple and efficient radiolabeling methods, undemanding in terms of equipment and infrastructure. To make them technically and economically accessible to the medical community and its patients, it appears mandatory to develop a procedure similar to the well-established kit-based ^99m^Tc chemistry. Already available commercial kits for the production of ^68^Ga radiopharmaceuticals have demonstrated the feasibility of using such an approach, thus paving the way for more kit-based ^68^Ga radiopharmaceuticals to be developed. This article discusses the development of ^68^Ga cold kit radiopharmacy, including technical issues, and regulatory aspects.

## Introduction

Today, medicine makes an extensive use of various imaging modalities based on different physical properties, including CT, ultrasonography, MRI, optical imaging or radionuclide-based single-photon emission computed tomography (SPECT), and PET, alone or in combination, to get anatomical and/or functional information in a non-invasive way. In particular, molecular imaging, always making use of more precise contrast agents, enables the visualization, characterization, and measurement of biological processes at molecular and cellular levels in humans and other living systems (1). Among available modalities, PET imaging has the advantage of unlimited depth penetration, very high sensitivity, capability to detect early changes at cellular or even molecular level, and superior resolution and quantification compared to SPECT (2–5). This type of examination allows for functional exploration of biological processes in order to obtain a diagnosis, prognosis, follow-up, or preselection for targeted therapy depending on the drug used (6–8). PET imaging and its hybrid derivatives PET/CT and the more recent PET/MR have had a tremendous impact on patient management, and have now become the new standard for functional imaging, both in oncology and non-oncological setting (9–14).

Positron emission tomography (PET) imaging is currently dominated by ^18^F-fluorinated radiotracers, and particularly the glucose analog ^18^F-fluorodeoxyglucose ([^18^F]-FDG), covering about 90% of PET scans in oncology, neurology, and cardiology. This tracer has found wide applications, taking advantage of increased glucose metabolic rates under several conditions, especially most cancers (2, 15). However, because of its mechanism of action, [^18^F]-FDG lacks specificity and cannot differentiate between a tumor-associated high metabolic rate and one due to infection or inflammation. Moreover, some tumors are non-FDG-avid and, thus, can hardly be imaged by it (16). This is the case, for instance, of early-stage prostate cancer and some types of gastric cancers (17, 18). This and a better understanding of biological mechanisms underlying metabolic and pathogenic pathways coupled with progress in radiochemistry have stimulated the development of novel PET radiopharmaceuticals (19–25). Initially, PET radiotracers were small endogenous molecules in which one atom was replaced with an equivalent or similar positron-emitting atom (^11^C, ^13^N, ^18^F, and ^124^I), thus minimally altering their *in vivo* behavior. With the expansion of medical applications, the quest for new PET nuclides has extended to radiometals, such as ^64^Cu, ^68^Ga, and ^89^Zr, to cite the most prominent ones (25–27).

Gallium-68 belongs to the family of post-transition metals including, among others, indium-111 and thallium-201. It decays to 89% by positron emission and to 11% *via* electron capture, with average positron energy per disintegration of 740 keV (E_β + *max*_ = 1.899 keV). Its 67.8-min physical half-life is compatible with the pharmacokinetics of most radiopharmaceuticals of low molecular weight, such as small organic molecules, peptides, or even antibody fragments, and oligonucleotides. It allows comfortable use from radiolabeling of PET tracers to acquisition of PET images. Another advantage of its short half-life is limited irradiation of a patient for an injected activity compatible with good PET image quality. In addition, gallium-68 can be conveniently obtained from a germanium-68/gallium-68 (^68^Ge/^68^Ga) generator, which can be used within a nuclear medicine department for 1 year thanks to the long half-life of the parent element (t_1/2_ Ge = 270.8 days). Besides, possible combination with ^90^Y, ^177^Lu, or, more recently, ^225^Ac to form a theranostic pair is another valuable feature (28). All these advantages make gallium-68 a powerful alternative to fluorine-18 (29), and have stirred an ever-increasing interest in ^68^Ga-based radiopharmacy and the number of patents pertaining to gallium-68 issued over the last decade (30). Spurred by the clinical and commercial success of ^68^Ga-labeled somatostatin analogs and PSMA ligands, numerous ^68^Ga-labeled agents have been reported, with some encouraging preclinical and clinical outcomes (31–36). ^68^Ga has even been described as a potential PET surrogate for ^99m^Tc, the workhorse for SPECT imaging (31, 37). Like the latter, it is expected that the development of kit-based ^68^Ga radiopharmacy would further expand its clinical usefulness by simplifying and reducing investment costs necessary for automated procedures while still respecting good radiopharmacy practice.

## ^68^Ga Production

### ^68^Ge/^68^Ga Generators

One of the main advantages of ^68^Ga is its cyclotron-independent mode of production. It is conveniently produced “on-demand” with a ^68^Ge/^68^Ga generator, in a similar way to the well-known ^99^Mo/^99m^Tc generator (38). ^68^Ga radiotracers can, thus, be available worldwide in a flexible way, even in centers far away from cyclotrons or production sites. With the secular equilibrium between the parent radionuclide ^68^Ge and its daughter ^68^Ga, the maximum theoretical activity generated is reached 14.1 h after last elution. However, after a time equal to three half-lives of ^68^Ga, or 3.4 h, nearly 91% of the maximum theoretical activity has already been generated. This allows, if necessary in clinical use, for an elution approximately every 4 h (up to 3 elutions/day), depending on the initial activity of the generator and its age (39).

The production of ^68^Ga from ^68^Ge has been described since the 1950s, and the first generator dates from 1960 and relied on a liquid/liquid extraction process (40). This extraction process was quickly supplanted by solid/liquid extraction *via* an ion exchange resin (41). The stationary phase (matrix) selectively retains Ge^4+^ ions while facilitating the elution of Ga^3+^ ions, which have chemical properties sufficiently different to allow several various methods for efficient separation (38). Currently, several generators are commercially available. They all consist of a solid matrix and are eluted with an HCl solution to obtain ^68^Ga^3+^. These generators differ from each other by the composition of their matrix, which is either inorganic or organic, and the concentration of HCl used for elution (ranging from 0.01 M for nano-zirconia matrix to 1 M for tin dioxide matrix) (39). As a result, they each have specific characteristics, which represent a major difference with ^99^Mo/^99m^Tc generators and one of the foremost challenges to develop cold kit formulations. Up to recently developed GMP-generators, obtained ^68^Ga eluate was not directly amenable to direct radiolabeling because of high volume, thus low ^68^Ga concentration, high [H^+^] concentration, ^68^Ge breakthrough, and presence of other potentially competing metallic cation impurities. Different elution procedures and Post-processing methods include: eluate fractionation, anion exchange, cation exchange, and a combination thereof (39, 42–44).

**Figure 1:**
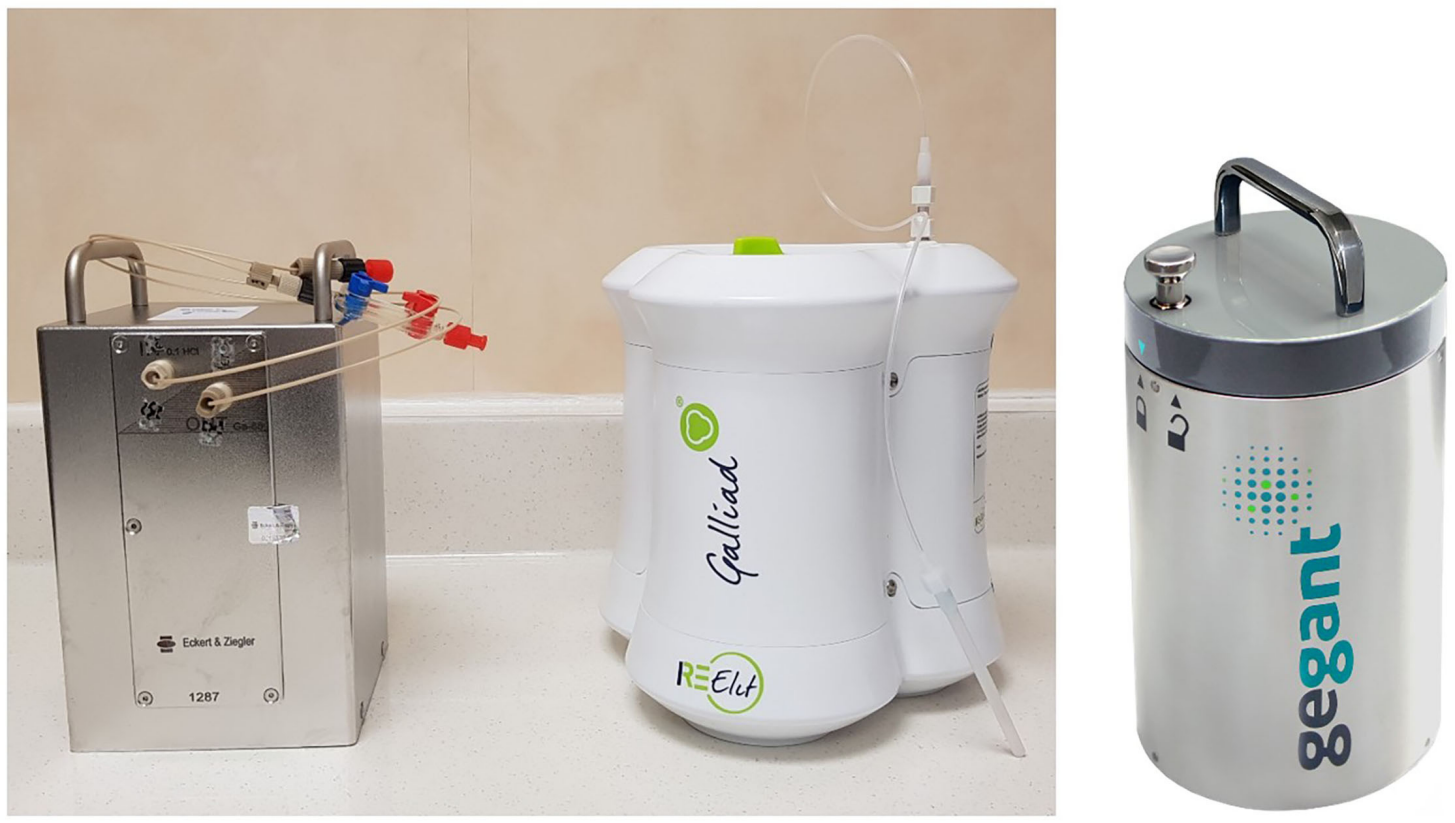
Radiopharmaceutical grade germanium-68 (^68^Ge)/gallium-68 (^68^Ga) generators from left to right: Galliapharm®, Galli Ad®, and GeGant®.

At this time, two TiO_2_-based ^68^Ge/^68^Ga generators are available for human clinical use, with marketing authorizations from the American Food and Drug Administration (FDA) and the European Medicines Agency (EMA): GalliaPharm® (Eckert & Ziegler AG, Berlin, Germany) and GalliAd® (IRE Elit, Fleurus, Belgium). The ^68^Ge/^68^Ga generator (GeGrant®) of ITG (Garching, Germany), with a dodecylgallate-modified SiO_2_ resin, has recently been granted a Type II Drug Master File from the FDA (Figure 1). Eluates of these generators comply with Pharmacopeia and can be used as is, without the need for post-processing, notably for reconstitution of cold kits.

### Cyclotron Production

Although ^68^Ge/^68^Ga-generators represent a convenient option for PET molecular imaging, there remains several limitations. First, the cost of generators can be high, depending on the supplier and/or the grade, and is only amortized if a sufficient amount of exams is performed. Second, because of constant increase in demand in recent years, there was shortage in generators from 2017 to 2018, which posed a threat on the development of clinical use of ^68^Ga-based imaging agents (45). Other supply issues are anticipated in the future, with increasing demand for ^68^Ga-imaging, particularly when PSMA imaging agents are approved and become reimbursable (46). Production capacities have been increased and secured. They will, non-etheless, remain limited and may not fulfill the global demand, with a maximum of about 3 preparations of 3 patient doses per day. Alternative production routes have, thus, been investigated (47). In this context, production of ^68^Ga with existing medical cyclotrons looks particularly attractive. Indeed, the global worldwide network of medical cyclotrons has quickly expanded over the last years.

Production of ^68^Ga in cyclotrons is usually done *via* the ^68^Zn(p, n)^68^Ga reaction., This reaction has high cross-section in the energy range of 11–14 MeV; thus, it is easily accessible in small medical cyclotrons (< 20 MeV). Either solid or liquid targets can be used, both having their advantages and limitations. For instance, with solid targetry, it is possible to obtain high amounts of ^68^GaCl_3_, up to 194 GBq, but with complex and expensive infrastructures (target holder, cooling, target transfer, and target processing) (48, 49). Utilization of a liquid target (dissolution of ^68^Zn in nitric acid) leads to lower amounts of radioactive material but offers a cost-effective alternative to solid targets, needing less investments, notably for ^18^F-producing cyclotrons that would like to implement ^68^Ga production. This looks particularly suited for small on-site production within hospital cyclotron facilities (50, 51). Main disadvantages of cyclotron production are the co-production of other radionuclides (potentially non-removable, since they possess the same chemistry like ^66^Ga and ^67^Ga) and the presence of metal impurities that may perturb the radiolabeling reaction of ^68^GaCl_3_. This necessitates complex target design and purification procedures, and is particularly true for solid targets (52). Because of growing interest in accelerator-produced ^68^GaCl_3_, the latter now has a European Pharmacopeia monograph (monograph 3109) (53). Cyclotron-produced ^68^Ga, either with solid or liquid targets, has reached the clinic (51, 54). No differences were observed in the quality of studies whether ^68^Ga was produced from a cyclotron or from a generator.

## Gallium Chemistry

Gallium is located in group 13 in the 4th period of the Periodic Table, with the electronic configuration [Ar]3d^10^4s^2^4p^1^. In aqueous solution, only the +III oxidation state is stable. The Ga^3+^ cation is a hard Lewis acid (pKa = 2,6), because of its high cation density and short ionic radius (62 pm), with close coordination chemistry to Fe(III), which has a similar ionic radius (65 pm) and same ionic charge (55). It is an electron acceptor. It has a d^10^ electronic configuration allowing it to accept different numbers of coordination, mostly 6, but 4 or 5 are also possible. It will preferentially coordinate with hard bases (electron donor species, with high electronegativity), that is to say ligands containing nitrogen, oxygen or, to a lesser extent, sulfur (such as carboxylate, phosphonate, hydroxamate, amines, thiolates, and phenolates groups), to form thermodynamically stable complexes (37, 56). However, the ionic form Ga^3+^ is only stable in a very acidic medium below pH 3. Indeed, at pH above 3, water acts as a weak ligand and gallium associates with hydroxide groups as described below:

Ga^3+^ + OH^−^ ↔ [Ga(OH)]^2+^ + OH^−^ ↔ [Ga(OH)^2^]^+^ + OH^−^ ↔ [Ga(OH)_3_] + OH^−^ ↔ [Ga(OH)_4_]^−^

The prevalent species formed at pH 3-7, [Ga(OH)_3_], is insoluble, and gallium is no longer available for a complexation reaction. At basic pH (> 7), gallium forms the gallate ion [Ga(OH)_4_]^−^ which is soluble. However, gallium remains unavailable for any complexation reaction (57). Hydrolysis and formation of insoluble hydroxides in the preparation of ^68^Ga radiopharmaceuticals remain a problem that can be circumvented by using weak, stabilizing ligands such as citrate, acetate, oxalate, and HEPES (2-[4-(2-hydroxyethyl)piperazin-1-yl]ethanesulfonic acid) (58). The latter, acting as both a buffer and a weak chelating ligand, has demonstrated superior performance when radiolabeling with ^68^Ga (59). There are, however, several limitations regarding its use pertaining to its potential toxicity for human use (60). For this, reason, acetate buffers are usually preferred.

The design of most ^68^Ga-labeled radiotracers is based on the use of bifunctional chelating agents. These compounds are capable of both coordinating the radiometal on one side and covalently conjugating to carrier molecules (i.e., a peptide) by an appropriate functional group on the other side. Bifunctional chelators must meet several criteria for the development of ^68^Ga-based radiopharmaceuticals:

- Their binding to a vector molecule must not alter their complexation to the metal. Chelation must be rapid and effective.
- Conversely, their binding to the vector molecule must not disturb the chemical characteristics, and therefore, its pharmacodynamic parameters *in vivo*. The size and charge of chelates can change the affinity of the tracer for its receptor. Lipophilicity may also interfere with the elimination of radiopharmaceutical drugs.
- They must be stable, with respect to hydrolysis, to avoid formation of hydroxides.
- The obtained chelate must be kinetically stable against demetallation, at physiological pH, and in the presence of other cations present in the serum (Ca^2+^, Zn^2+^, Mg^2+^).
- ^68^Ga complexes must be more stable than ^68^Ga-transferrin complexes to avoid transchelation because transferrin has two binding sites of metal ions. As already mentioned, gallium has a strong similarity to iron, from a chemical point of view. *In vivo*, iron is transported by transferrin mainly at the hepatic level. This protein tends to remove gallium from its ligand if the complex is of low affinity, which explains the hepatic binding of gallium. Transferrin, therefore, constitutes a sort of “reference” during tests of gallium-68 chelating agents [mean pGa is 19.7, and log (β) is 20.3 and 19.3 on each of the two sites of binding carried by transferrin] (61).

**Figure 2:**
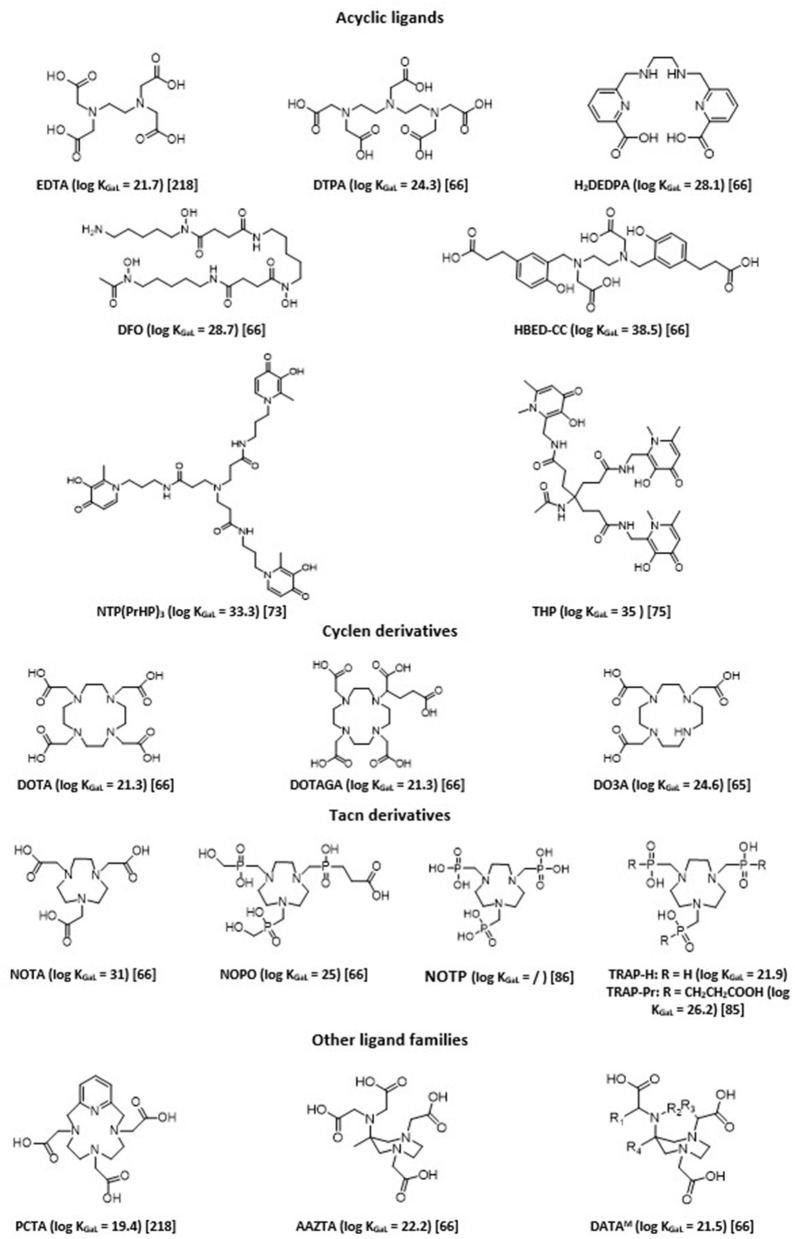
Chelates commonly used with ^68^Ga.

A strong coordination of chelating agents is always necessary to obtain sufficient stability. Therefore, ligands that form highly stable complexes with Ga^3+^ ions are hexadentate. They sequester Ga^3+^ using its maximum coordination number (*n* = 6). The chemistry of six-coordinate Ga(III) complexes has been comprehensively reviewed (62). Chelation with gallium-68 has been extensively studied, allowing fine tuning of obtained ^68^Ga imaging agents in terms of charge and lipophilicity, and thus their pharmacokinetics and biodistribution profiles (27, 29, 31, 37, 63–67). There are two main classes of bifunctional chelating agents: macrocyclic and acyclic ligands. In general, acyclic chelating complexes are less inert than macrocycles of comparable stability, but they have higher complexation kinetics than cyclic chelating agents. Overall, a large number of labeling carried out with these acyclic molecules can be done at room temperature rather quickly and with good yield. Examples of chelating agents used with ^68^Ga are given in Figure 2.

Acyclic polyaminopolycarboxylate ligands, such as ethylenediaminetetraacetic acid (EDTA) and diethylenetriaminepentaacetic acid (DTPA), were among the first chelators to be used with ^68^Ga for blood cell and renal imaging (68, 69). They have also been used as bifunctional chelators. Unfortunately, most of these complexes showed low stability *in vivo*, since such anionic complexes tend to undergo acid- or cation-promoted demetallation. More stable complexes were subsequently synthesized. Among the most widely described ligands investigated, one can cite desferrioxamine (DFO) and its derivatives, which form a stable, neutral complex by coordinating Ga(III) through three hydroxamate groups (70). H_2_DEDPA (6,6'-(ethane-1,2diylbis(azanediyl))bis(methylene)dipicolinic acid) has good characteristics in terms of labeling parameters, quantitatively chelating ^68^Ga^3+^within 10 min at RT, with ligand concentrations as low as 10^−7^ M (71). Its *in vivo* clearance, however, appears slow (72). Tripodal compounds of the 3,4-hydroxypyridinone family, such as *tris*(hydroxypyridinone) (*aka* THP or CP256) and NTP(PrHP)_3_, are also promising chelators for ^68^Ga, with quick chelating ability and high *in vivo* stability (73, 74). They also allow for complexation of ^68^Ga^3+^ above pH 5 at ambient temperature and can be conjugated to peptides or other targeting molecules. THP shows remarkably high affinity for Ga^3+^ ion [log (β) of 35 with Ga^3+^, pGa of 30]. Maybe its main drawback is its sensitivity for metallic impurities commonly present in ^68^Ge/^68^Ga generators or with the material used, such as Al^3+^, Fe^3+^, and Ti^4+^, although it was shown to be less sensible than DOTA or NOTA to other metallic impurities possibly present (Zn^2+^, Cr^3+^, Ni^2+^, and Pb^2+^) (75). Several ^68^Ga hydroxypyridinone-based bioconjugates with biomolecules of interest (PSMA, TATE, RGD…) have been reported in the literature (76). The latter two examples ([^68^Ga]Ga-THP-TATE and [^68^Ga]Ga-THP-RGD_3_) led to inferior biodistribution results when compared with their DOTA-counterparts, while [^68^Ga]Ga-THP-PSMA led to positive outcomes, with tumor imaging characteristics comparable to the current “gold standard” [^68^Ga]Ga-PSMA-11 (77).

Finally, HBED (N,N'-bis(2-hydroxybenzyl)ethylenediamine-N,N'-diacetic acid) is currently considered the gold standard for acyclic chelating agents. HBED is a related derivative of EDTA, which was first described in the 1960s (78). It has a strong affinity for Ga^3+^ with a pGa of 28.6. It is characterized by rapid complexation reaction with Ga^3+^ with very good yields, and its stability in serum is comparable to that of NOTA, which sets it apart from the majority of acyclic chelators. The complexation reaction is rapid even at ambient temperature; it is, therefore, a particularly advantageous ligand for radiolabeling of thermolabile molecules. Among its functionalized derivatives, HBED-CC (N,N'-bis[2-hydroxy-5-(carboxyethyl)benzyl]ethylenediamine-N,N'-diacetic acid) has been particularly studied and used. It presents bifunctionalization with two carboxyl arms in the phenyl rings, which allows its conjugation to multiple vector molecules (79–82). HBED derivatives have a coordination of six and engage four oxygen atoms (the two hydroxyls of the phenyl rings and those of the two acetic functions) and two nitrogen atoms (those of the ethylenediamine function) in the complexation of Ga^3+^. This complexation causes a modification of the conformation of the ligand that can form three distinct diastereomers each having two enantiomers (67). Heating is necessary to increase the formation of the most thermodynamically favored isomer, since configuration might impact the biological behavior of the radiotracer.

Poly-aza-macrocycles derived from cyclen (1,4,7,10-tetraazacyclododecane) and TACN (1,4,7-triazacyclononane) are currently the most studied ligands. The metal is trapped within a cage-like structure formed by the ligand. It is possible to increase the number of interactions with the radionuclide by adding electron donor groups on the side chains of the chelating agents, which improves the stability of the complex and may eventually provide additional functionalization sites useful for conjugation to the vector molecule. Cyclic chelating agents exhibit good complexation inertia, with little demetallation phenomena seen *in vivo*; hence their significant use (37). DOTA, a cyclen substituted by four carboxylic arms (1,4,7,10-tetraazacyclododecane-1,4,7,10-tetraacetic acid), is the most common gallium chelator (83). In the case of ^68^Ga, the maximum coordination number is 6; therefore, only two carboxyl arms are used for complexation; thus, one of the carboxyl groups in DOTA is often transformed and used to allow for conjugation to the vector in the form of a very low complexing activated ester. This decreases the denticity of the chelator, which often causes decrease in stability, especially for metals with a coordination number of 8. Despite an average stability with Ga [log (β) of 21.33 with Ga^3+^ and pGa of 15.3], close to those of the Ga-transferrin complex, DOTA has great inertia kinetic. However, this involves a slow complexation reaction. Radiolabeling protocols necessitate heating at more than 90°C for several minutes to obtain a yield >90%. This limits its use on heat-resistant molecules. Many DOTA derivatives have, thus, been developed to address these issues (increase the denticity of DOTA and/or allow labeling at room temperature): p-SCN-Bn-DOTA, C-DOTA, DOTAGA, DOTASA, CB-DO2A, TCMC… (37, 84).

Derived from TACN, NOTA (1,4,7-triazacyclononane-1,4,7-triacetic acid) is a hexadentate chelating agent whose N_3_O_3_ core provides high thermodynamic stability [log (β) of 30.98 and pGa of 26.4]. This is due to the size of its cycle, which is more adapted to the ionic radius of Ga^3+^ compared to DOTA. NOTA engages its three carboxylate arms in the complexation of Ga^3+^ and presents a neutral charge at physiological pH. In this configuration, none of the arms are available for functionalization. If one of its carboxylate arms is mobilized to create a direct bond to a vector, the thermodynamic stability of the complex is compromised, because the optimal coordination of 6 of Ga^3+^ is no longer possible. In addition, the complex becomes positively charged, which can alter the biological properties of the vector (85). Different functionalized derivatives have been proposed to be conjugated to peptides while preserving the maximum denticity of the NOTA, with, in particular, functionalization on a carbon between a carboxyl and a nitrogen of the ring (i.e., NODAGA). Phosphinic and phosphonic acids functionalized polyazacycloalkanes, such as NOTP, NOPO, and TRAP derivatives, and showed themselves as powerful bifunctional chelators for the preparation of ^68^Ga-based targeted imaging probes, forming very stable complexes (85–87). They demonstrate significantly improved selectivity for Ga^3+^ ions, and, hence, are less sensitive to potential metal ion contaminants (88, 89). These triazacyclononane-triphosphinates ligands also have the advantage of chelating ^68^Ga at very low concentrations and in strongly acidic media. They, thus, would permit labeling directly from an acidic generator eluate, which could ease automation or kit formulation (90).

Recently, hybrid chelators, combining fast complexation kinetics under mild conditions of acyclic ligands with the prolonged complex stability of the macrocyclic ones, have been introduced. H_4_AAZTA (N,N',N”,N”(6-amino-6-methylperhydro-1,4-diazepine)-tetraacetic acid) demonstrates good qualities, especially regarding its simple conditions of use, and high kinetic inertness and thermodynamic stability of the resulting complex, with a pGa of 22.4 (91, 92). It is, however, not totally stable against serum and competitors. Other more stable chelators based on the 6-amino-diazepine scaffold, have been developed, DATA^X^ chelators (93). They allow for quantitative labeling with ^68^Ga with favorable kinetics at ambient temperature, and within a large range of pH (4-7). Besides, formed ^68^Ga-chelates display excellent stability toward transchelation (94). A further advantage of this type of ligands is their suitability to also chelate therapeutic ^177^Lu radionuclide (95).

Tsoniou et al. have performed an extensive side-by-side comparison with some of the most prominent chelators for ^68^Ga labeling (67). They demonstrated that NOTA, NOTP, TRAP, HBED, DFO, and THP were all efficiently and quickly labeled at near neutral pH, room temperature, and that with low chelator concentration, THP and DFO are the most effective under these conditions. They are, thus, ideal candidates for instant kit preparation. Among TACN ligands, NOTP appeared as probably the most promising one for labeling under mild conditions (ambient temperature, near-neutral pH). The Ga(NOTP)^3−^ complex, however, appears to be more prone to hydrolysis under basic conditions (86). So far, studies investigating its potential usefulness as a bifunctional chelating agent are scarce. Unfortunately, AAZTA and DATA derivatives were not included in the comparative study by Tsoniou et al. However, a direct comparison between DATA chelators and NOTA demonstrated the potential superiority of the DATA family over NOTA (94). DATA chelators represent, thus, another potential ideal candidate for instant kit preparation. Care should be taken when choosing the chelator for it can have a non-negligible influence on final radiotracer behavior (96). Choice of optimal bifunctional chelator candidate for the preparation of ^68^Ga imaging agents has to be made based on systematic comparison among different chelators coupled with the same targeting moiety. The best candidate is the one with the best compromise between radiochemistry considerations and pharmacologic parameters, determined by *in vitro* and subsequently *in vivo* studies. For instance, Varasteh et al. investigated the influence of four different macrocyclic chelators on a bombesin analog targeting properties (97), while Renard et al. evaluated seven different chelators coupled to a neurotensin receptor 1 antagonist (98). Other examples of such a strategy also include comparison of [^68^Ga]Ga-THP-TATE with [^68^Ga]Ga-DOTATATE and [^68^Ga]Ga-AAZTA^5^-PSMA-617 with [^68^Ga]Ga-DOTA-PSMA-617 (99, 100).

## Automation

Radiolabeling developed in preclinical research often starts with a manual optimization phase of reaction conditions. However, in order to improve the radiation protection of personnel and facilitate transfer for routine clinical use, automated modules have been developed. They are piloted by software that allows to remotely control the sequence of operations required for labeling. They allow higher reproducibility and robustness, especially in critical stages such as elution of generator and purification. Parameters are checked continuously, and summary data are plotted and stored. It, thus, enables to face the increasing regulatory issues required for hospital-based preparation of PET radiopharmaceuticals (101, 102). Transfer to other institutions is also facilitated with the use of standardized and validated technologies and procedures.

**Figure 3:**
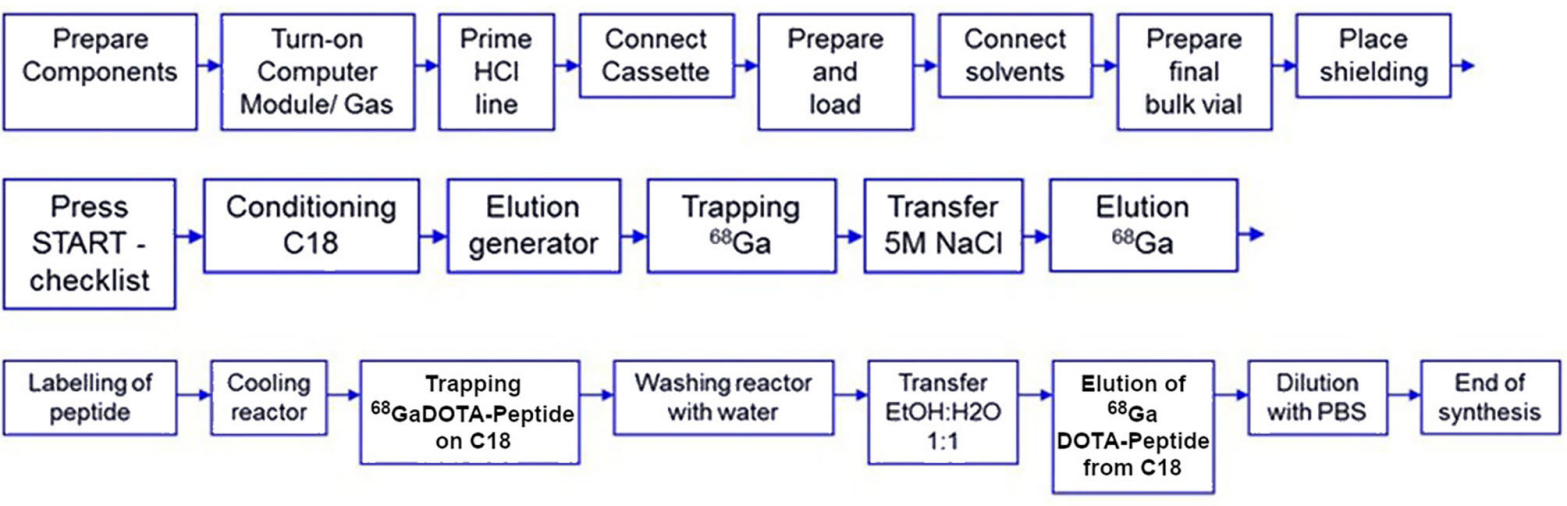
Typical automated procedure for ^68^Ga labeling of a DOTA peptide [adapted from (106)].

Several semi-automated and automated systems have been developed, either in-house built or commercially available products, combining generator elution and post-processing, ^68^Ga-radiolabeling, and purification of the final ^68^Ga radiotracer. It has to be noted that all post-processing approaches (fractionation, anionic and cationic purification) have been adopted in commercially available automated systems. These apparatuses can be classified into two types, depending on the technology used: fixed tubing or single-use disposable cassette (103). Both approaches have their respective pros and cons. Fixed tubing systems were the first to be developed and are still extensively used in preclinical research (104). They are indeed extremely flexible. Modification at will of sequence parameters to optimize labeling is possible. They are also cost-effective. On the other hand, they require stringent cleaning and disinfection procedures to maintain sterility, and cross-contamination cannot be excluded if several radiotracers are prepared using the same module. The cassette system approach presents with the advantage of using sterile single-use cassettes. It, therefore, offers enhanced microbiological safety and eliminates risks of cross-contamination, accordingly better complying with cGMP requirements. Multiple syntheses can easily be achieved, even for different tracers and/or radionuclides, by simply changing the cassette (105). Cassette-based synthesizers, nonetheless, have some downsides. They allow less flexibility than fixed tubing ones. Moreover, using disposable cassettes has a non-negligible cost and generates reliance on cassette manufacturers. New, validated cassette development strongly depends on marketing considerations. Presently, there is a number of sterile GMP cassettes to produce ^68^Ga-radiotracers for targeted imaging of somatostatin and chemokine receptors or prostate-specific membrane antigen, commercially available from all automated synthesizer manufacturers. Figure 3 presents a typical automated procedure for ^68^Ga labeling of a DOTA-peptide.

Future developments in automation might be using microfluidic, in a dose-on-demand strategy. This approach would enable reducing the size of modules as well as cost to produce a dose (107, 108). Care should be taken, though, of possible surface effects and radiolysis owing to high radionuclide concentration. Research on that domain is still very preliminary and has not reached clinical use.

## Cold Kits

As mentioned above, automated preparation of ^68^Ga imaging agents generates substantial investments (in hot cells, synthesis modules, and quality control equipment), which may not be easily accessible to small-scale radiopharmacies. This could hamper the widespread use of ^68^Ga-PET imaging. As a consequence, interest in the development of cold kit formulations, as a simpler and cheaper approach, has increased steeply in recent years (94, 109–111).

### Technical Challenges

#### Generator Eluate

The synthesis of ^99m^Tc radiopharmaceuticals is easily carried out using commercial kits, containing a reducing agent (most often tin chloride), a ligand (intended to form the desired complex or an intermediate complex), and, possibly, other components (buffer, stabilizing agents, cryoprotectant, excipients…). Kits are lyophilized and sealed, and are, therefore, sterile, pyrogen-free, and can be stored for a long time. This approach offers convenience and ease of use, simply adding the required activity in a certain volume of eluate, with or without the need for heating (112). Technetium-99m issued from ^99^Mo/^99m^Tc is eluted under the form of sodium pertechnetate, with a sterile and pyrogen-free solution of sodium chloride. This solution can be used directly for the preparation of radiotracers.

**Figure 4:**
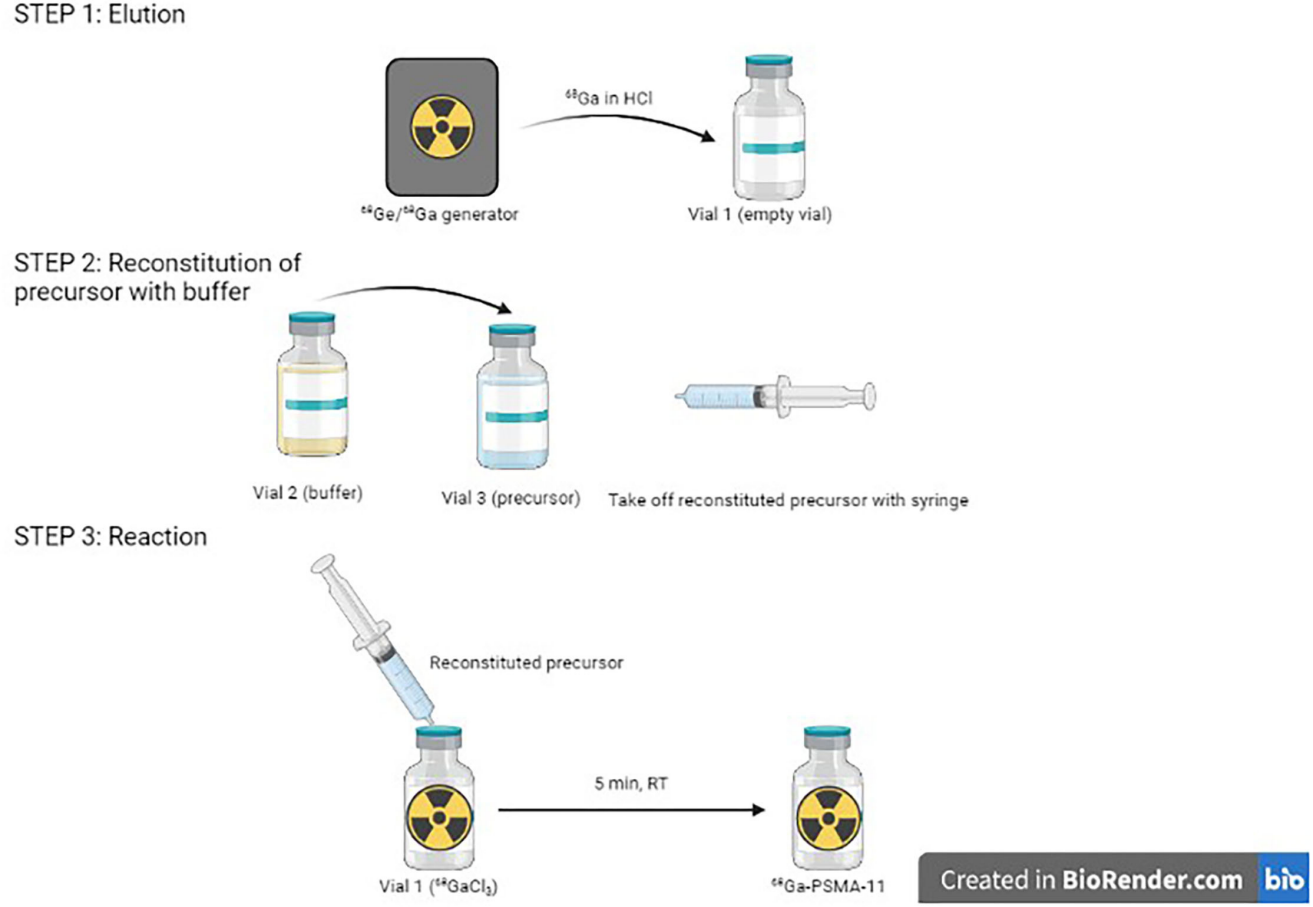
Procedure for the preparation of ^68^Ga-HBED-CC-PSMA-11 (Telix Pharmaceuticals).

On the other hand, ^68^Ga eluate is a strongly acidic solution, and is obtained using HCl in various amounts and various molarities, depending on generator type. It can also come from a cyclotron, with still other characteristics. This is a challenge for the formulation of kits. Indeed, addition of highly varying ^68^Ga eluates into a fixed buffer amount inevitably leads to pH variations of the final solution. However, apart from use with few chelating agents, gallium chemistry is very pH-sensitive. It is, thus, essential to buffer the solution within an adequate pH range and stabilize ^68^Ga^3+^ ion (58). One proposed solution is to prepare 1-vial generator-specific kits, with a fixed amount of buffer in the freeze-dried vial to enable to reach optimal pH in function of HCl volume and molarity required to elute the generator. For instance, to develop a kit-based [^68^Ga]Ga-DOTATOC preparation, Asti et al. prepared kits with either 180 or 380 μl of 1.5 M sodium formate for use with, respectively, ITG (eluted with 0.05 M HCl) and Eckert & Ziegler (eluted with 0.1 M HCl) generators, to maintain the reaction pH around 3.3 (113). Vats et al. developed three different formulations of a [^68^Ga]Ga-RM2 kit to be used with ITG, Eckert & Ziegler, and iThemba generators (Faure, South Africa) (114). With this solution not being very practical, another proposed possibility has been to develop 2-vial kit formulations similar, for instance, to [^99m^Tc]Tc-mercaptoacetyltriglycine ([^99m^Tc]-Mertiatide). One vial contains the lyophilized ligand and excipients, and the other the buffer solution. This way, it is possible to adjust the necessary amount of buffer to reach suitable pH for the labeling to proceed. This can be exemplified by [^68^Ga]Ga-DOTATOC and [^68^Ga]Ga-DOTATATE kit formulations (106). The kit for the preparation of [^68^Ga]Ga-HBED-CC-PSMA-11, developed by ANMI (now part of Telix Pharmaceuticals, Melbourne, Australia), is also provided with a third sterile vacuumed vial for initial elution before addition of the buffer-dissolved precursor (Figure 4).

**Figure 5:**
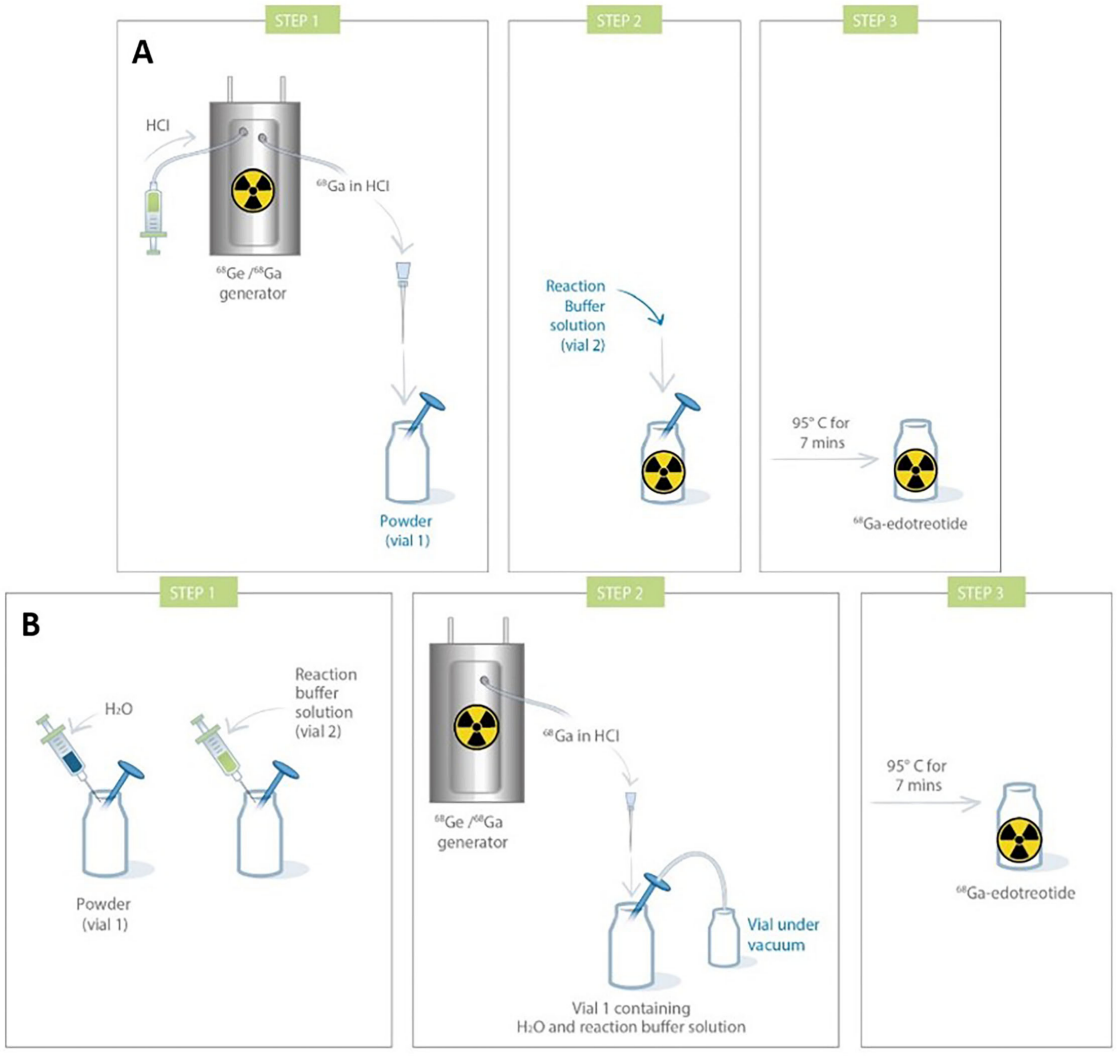
Comparison of procedures for Somakit TOC® labeling with 2 different commercial generators [**(A)**. Galliapharm (Eckert & Ziegler) GalliAd (IRE Elit); **(B)**. GalliAd® and IRE Elit) (116).

Most of the kits developed up to now have been validated with one generator model, usually dependent upon geographic and/or financial availability. There is currently only one example of a cold kit used with an accelerator-produced ^68^GaCl_3_ eluate (115). When using different generators, besides tweaking the formulation, it might also be required to adapt the radiolabeling protocol. For instance, [^68^Ga]Ga-DOTATOC (Somakit TOC® Novartis) was initially developed for use with a Galliapharm® (Eckert & Ziegler, Basel, Switzerland) generator, at a time when the latter was the only authorized generator for human use. It was, thus, designed to be reconstituted with 5 ml of ^68^Ga-eluate. To be used with the newly introduced GalliAd® (IRE Elit) generator and its 1.1-ml eluate, a different procedure had to be validated by, notably, dilution with sterile water (Figure 5). When preparing [^68^Ga]Ga-DOTAGA-TOC and [^68^Ga]Ga-DOTAGA-TATE using an Eckert & Ziegler generator, Satpati et al. passed the generator eluate through a Strata™ X-C cation exchange column, which was then eluted with a 500-μl acetone/HCl mixture (97.6%/0.02 M) before the addition of the purified eluate to the kit vial (117). Using an ITG generator, the eluate was directly added to the kit vial. Tuning the molarity of the buffer is also a way to enhance the robustness of buffering toward HCl. In another study by Satpati et al., they reported that increasing sodium acetate buffer from 0.5 to 1.5 M allowed to yield suitable pH conditions either with an Eckert & Ziegler generator or with an ITG generator when preparing [^68^Ga]Ga-DKFZ-PSMA-11 (118). Using even a very small amount (250 μL) of 2-M acetate buffer, on the other hand, led to very high pH of 5.5, even for the most acidic E&Z generator.

#### Chelator

Use of robust, gallium-specific, bifunctional chelators is another step toward kit-based ^68^Ga-radiopharmacy. Promising ligands, like macrocyclic NOTA phosphinic derivatives, acyclic HBED-CC and THP, and hybrid DATA chelators, allow for fast and easy complexation with ^68^Ga under mild conditions, which is amenable to instant kit-type procedure. Several kits using these chelators have been reported in the literature, the most notable being HBED-CC-PSMA-11 used in two commercially available cold kits for prostate cancer imaging (Illumet™/Illuccix®, Telix Pharmaceuticals, and IsoPROTrace-11®, Isotopia, Petah Tikva, Israel) (119). The main difference between both kits is that the first kit consists of three vials, while the latter contains only one. PSMA binding motif lysine-urea-glutamate (KuE) has been attached to a THP chelator in yet another investigational kit, which was recently the object of a phase 2 trial (120). The same motif was also successfully grafted on DATA^5M^ and AAZTA^5^ chelators, but no kit formulation has been reported to date, although it is amenable to do (121).

Even though these chelators revealed themselves as successful in complexation of ^68^Ga in a fast and stable manner, they, with the possible exception of AAZTA/DATA chelators, unfortunately do not allow for complexation of other radiometals with higher coordination number, such as ^177^Lu, ^90^Y, and ^225^Ac. Thus, from a theranostics perspective, DOTA and its derivatives still remain the chelator of choice, even for ^68^Ga. Besides,^68^Ga-labeled DOTA-peptides generally demonstrate very good pharmacological properties. Kit formulation with DOTA/DOTAGA precursors is, therefore, still an active research area (113, 114, 117, 122).

#### Precursor

**Figure 6:**
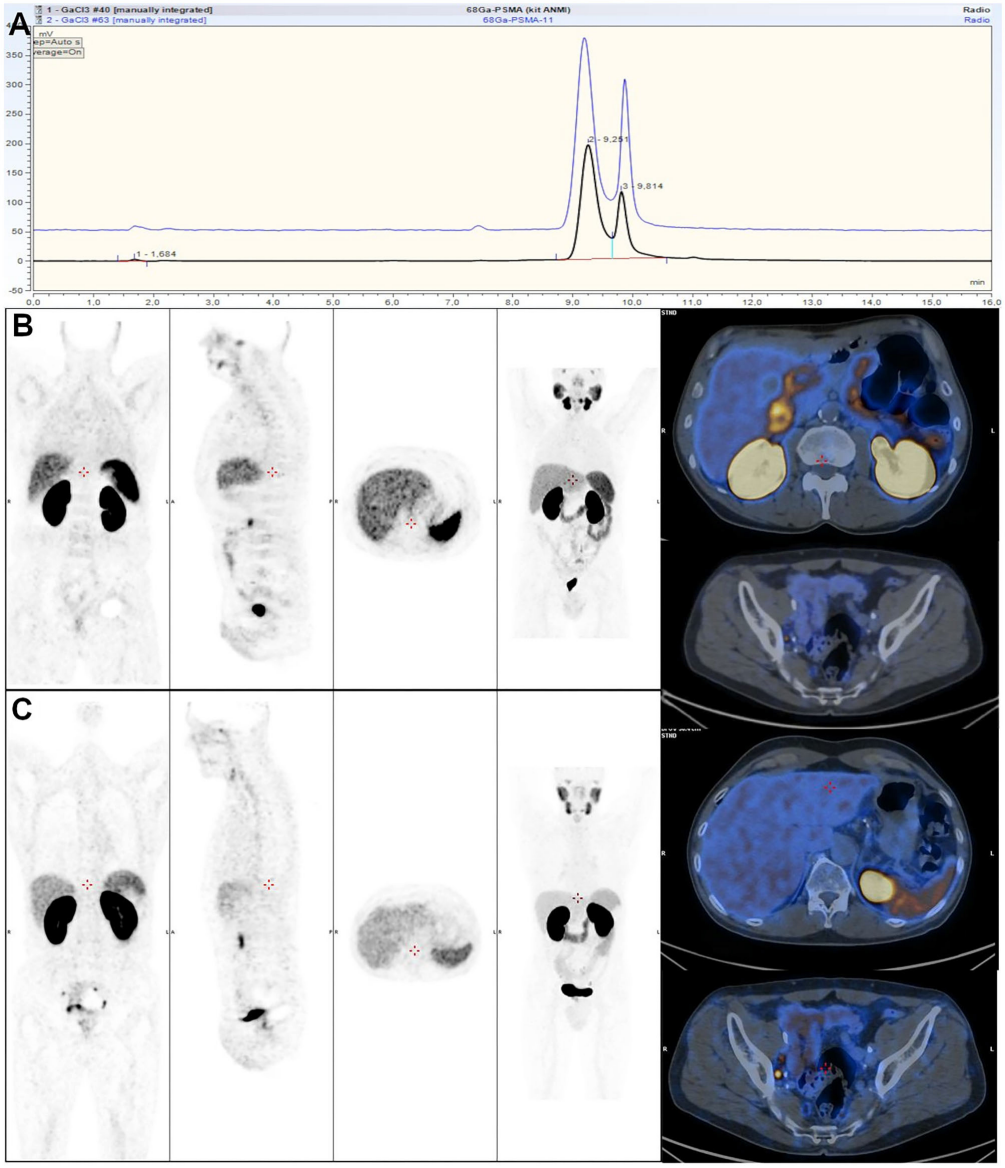
**(A)** Comparison of HPLC profiles of automated (blue) and cold kit-based (black) labeling of [^68^Ga]Ga-HBED-CC-PSMA-11. **(B,C)** Comparison of 2 ^68^Ga-PSMA-11 PET/CT acquisitions rated as good quality using ^68^Ga-PSMA-11 synthetized with sterile cold kit **(B)** and ^68^Ga-PSMA-11 synthetized with automated module **(C)**. **(B,C)** from (126).

To be sure that the reaction is complete and no post-labeling purification step is required, kits are generally prepared with higher ligand amount. For instance, 40 μG of a DOTATOC precursor is present in lyophilized kit formulation, while only 20–25 μg is necessary when [^68^Ga]Ga-DOTATOC is prepared manually or with an automated synthesizer (123). Likewise, 10 μg of a PSMA-11 precursor for automated process is used vs. 25 μg for an Illumet™ kit. This has to be optimized finely to prevent saturation of targeted receptors with unlabeled biomolecule. On the other hand, this, plus shorter production time and higher radiolabeling yield with the kit than with automated module synthesis, might allow for multiple patient preparations, although kits are usually recommended as single-dose preparations (118, 124, 125). Regarding outcomes of both processes, in terms of product characteristics, quality control gave comparable results (Figure 6A), and both methods are reliable and comply with Good Manufacturing Practices and European Pharmacopeia specifications (113, 124, 126, 127). In terms of clinical applications, a direct comparison on patients with prostate cancer found no significant differences in PET/CT image quality (Figures 6B,C) (126).

#### Kit-Based vs. Module Based Radiolabeling

Several studies discussed the respective pros and cons of cold kit-based preparation and module-based automated synthesis (126–128). To summarize, main advantages of the kit-based process are cost, shorter production time, simplicity of use, no purification needed, no EtOH content, and lower final volume. Main drawbacks are radiation exposure, higher amount of precursor usually needed, strong dependence on generator quality since there is no purification step, and few kits currently established and even fewer commercially available. To detail more precisely the cost efficiency of ^68^Ga cold kit labeling, besides savings on expensive synthesizer and its maintenance, and, according to local regulation, even the need for a shielded hot cell, cost per synthesis is significantly decreased. For instance, Kleynhans et al. calculated that one [^68^Ga]Ga-HBED-CC-PSMA-11 synthesis with a module and commercially available cassette and precursor costed 274 €, and that the cold-kit synthesis amounted for only 10 € with an in-house made kit (127). It should be noted that a commercial kit would be more expensive. In a study on [^68^Ga]Ga-RM2 synthesis, consumable costs for a synthesis was reported to be 282.1 vs. 65.7 € for a module and a kit, respectively, whereas the shorter production time and higher radiolabeling yield with the kit would allow for the injection of at least two additional patients (125). This gain in production time may also improve patient workflow, improving, again, cost efficiency.

Concerning radiation protection, there are only few direct comparisons between kit-based and module-based preparations reported in the literature. Frindel et al. compared [^68^Ga]Ga-DOTATOC kit preparation with automated [^68^Ga]Ga-DOTANOC labeling (129). Based on detector probes fixed on the first phalanx of each middle finger, they extrapolated extremity dose measurements for a 1.85-GBq generator at calibration. They reported 70 and 132 μSv for the automated labeling (left and right hand) and 179 and 152 μSv for the kit-based preparation. In the study by Kleynhans et al. on [^68^Ga]Ga-HBED-CC-PSMA-11 synthesis, whole-body exposure was found to be significantly lower for the automated synthesis with 2.05 ± 0.99 μSv vs. 14.32 ± 5.3 μSv per synthesis (127). However, automated synthesis was performed on a hot cell with 50-mm lead shielding, while the kit preparation was realized in a 3-mm lead-shielded class II biosafety cabinet, with a 10-mm lead shielded tabletop shield. When performing kit radiolabeling on a 50-mm lead hot cell, whole-body exposure was brought down to 2 ± 0.5 μSv per synthesis. Extremity dose was calculated to be 1.5 ± 0.4 mSv per kit synthesis. A major advantage of automated synthesis on radiation exposure was lower variation. For the kit labeling, radiation exposure was highly varying, depending on operator experience. Moreover, this can lead to radiation protection concerns in centers with high patient workflow. To circumvent this problem, several teams have investigated the possibility of partly or fully automating kit preparation. For instance, some teams eluted their ^68^Ge/^68^Ga generator with an automated module before reconstituting the kit vial (130). Revy et al. investigated both an automated and a semi-automated method for the labeling of kit-based DOTATOC formulation with ^68^Ga (131). Automated method led to longer preparation time, and, above all, possible product loss. Indeed, since the synthesizer they used, like most commercial synthesizers, does not possess a cooling block, transfer to another vial after the heating step was necessary to avoid overheating. The semi-automated process was developed as an alternative to product transfer with manual removal of the vial from the heating block. Semi-automated vs. manual kit labeling reduced significantly radiation exposure with a whole-body dose of 0.35 ± 0.19 vs. 0.98 ± 0.96 μSv/GBq and an extremity dose below the limit of quantification vs. 0.567 and 0.467 mSv/GBq (right and left hands). These methods are easily transposable to other cold kit preparations, especially those not requiring heating. Kit-based preparation of ^68^Ga-MAA (macroaggregated albumin) was conveniently automated using small Modular Lab EAZY (Eckert & Ziegler) (132). In front of this interest in the automation of kit radiolabeling, the industry has developed dedicated modules, like KitLab (Eckert & Ziegler) and MorGaNA (Tema Sinergie, Faenza, Italy) (133). These have the advantage of small footprint but have less flexibility than common automated modules.

### Regulatory Aspects

There are several articles that review the regulatory framework for radiopharmaceuticals in the US and Europe (101, 134–136). Radiopharmaceuticals are defined as medicinal products. In Europe, medicinal products are regulated in Directive 2001/83/EC (137), which also extends to cold kits, radionuclide generators, and radionuclide precursors (138). This implies that radiopharmaceuticals, and kits, need a marketing authorization, and a production process according to Good Manufacturing Practices (GMP), whose guidelines are specified in a separate Directive 2003/94/EC (139). Specific rules apply according to the status of the radiotracer. Investigational medicinal products and radiopharmaceuticals prepared “in-house” for local use (magistral/officinal preparation or compounding) are exempted from Directive 2001/83/EC (101, 140). Taking into account the particularities of radiopharmaceuticals, the EANM and its Radiopharmacy Committee have released specific guidelines, dubbed current Good Radiopharmacy Practice (cGRPP), for the production of radiopharmaceuticals in small-scale radiopharmacies, which do not cover commercial radiopharmaceuticals (141). For radiopharmaceuticals prepared from a licensed generator and a licensed kit, quality control should be realized according to the procedures described in the Summary of Product Characteristics (SmPC) for generator eluates and kit-based radiopharmaceuticals. When preparing from unlicensed components, procedures are more stringent to ensure maximum safety. When available, use of the appropriate European Pharmacopeia (Ph. Eur.) monograph or similar guidelines and documents published by the FDA is required. Implementation of a new radiopharmaceutical into pharmacopeias follows an extended procedure. For instance, [^68^Ga]Ga-PSMA-11 was granted a Ph. Eur. monograph in 2020 (monograph 3044) despite its extensive use for several years (53). Consequently, the majority of ^68^Ga-based imaging agents is not yet represented with their own monographs in the Pharmacopeias. In this case, general Ph. Eur. monograph “Radiopharmaceutical preparations” (monograph 0125) or closely related specific monographs (i.e., [^68^Ga]Ga-DOTATOC monograph, monograph 2482) can be used. Additionally, local authorities could demand more detailed quality control, even for licensed radiopharmaceuticals. The quality of the final radiopharmaceutical must fulfill all specifications given by the relevant legislation or Pharmacopeia no matter the synthesis route (manual, automated, or kit-based) (142).

A kit (or radiolabeling kit) is defined as “Any preparation to be reconstituted or combined with radionuclides in the final radiopharmaceutical, usually prior to its administration” (141). Cold kit-based radiolabeling is considered a closed procedure, consisting of preparation of a sterile radiopharmaceutical through the addition of a sterile eluate to a sterilized close vial containing a set of sterile, lyophilized ingredients via a system closed to the atmosphere. The final product is, thus, a sterile and pyrogen-free solution suitable for intravenous injection. It is of utmost importance to rigorously follow the instructions given by the manufacturer, especially regarding the maximum activity and volume of ^68^Ga eluate that is transferred to a kit vial (116). The European Pharmacopeia states that “the marketing authorization holder of a licensed kit is responsible to ensure compliance of the kit with the requirements of its marketing authorization, while the final user carries the responsibility for the quality of the preparation and the handling. If the given instructions are not strictly followed or if one or more components used for the reconstitution do not have marketing authorization, it is the responsibility of the final user to demonstrate that the quality of the final preparation is suitable for the intended use” (143). On its side, the FDA considers “cold kits to be finished drug products. Therefore, preparation of a radiopharmaceutical from the components of a cold kit according to FDA approved labeling is not compounding. However, if an ingredient is added, or if the cold kit is otherwise manipulated in a manner not considered a minor deviation, it would be considered compounding,” which could only be done by or under the supervision of an authorized radiopharmacist (144).

### ^68^Ga Cold Kits and Their Applications

An overview of the kits reported in the literature is given in Table 1.

**Table 1:** Overviewof cold kits reported in the literature for the preparation of gallium-68 (^68^Ga) imaging agents.

**^**68**^Ga-labeled molecule**	**Commercial name**	**Amount active substance**	**Number of vials**	**Eluate volume (mL)**	**Labeling conditions**	**References**
**Somatostatin analogs**							
DOTATOC	Somakit TOC®	40 μg	2	5	7–10 min, 95°C, pH = 3.2–3.8	(116)
DOTATATE	NETSPOT®	40 μg	2	5	7–10 min, 95°C, pH = 3.2–3.8	(145)
DOTAGATOC/DOTAGATATE	N/A	50 μg	1	0.5	5–10 min, 90°C, pH = 4	(117)
DATATOC	N/A	13 nmol	1		1–10 min, 23°C, pH = 4.2–4.9	(146)
NODAGA–JR11	N/A	75 μg	1	5	7 min, 90°C, pH = 4	(147)
**Prostate–specific membrane antigen ligands**							
PSMA-11	Illumet™	25 μg	3	1.1–5	5 min, RT, pH = 4–5	(148)
	IsoPROTrace-11®	10 μg	1	2.5	5 min, RT, pH = 4–5	(149)
	N/A	5 nmol (5 μg)	1	1	15 min, RT, pH = 4.0–4.5	(80)
	N/A	20 μg	1	1	10 min, 85°C, pH = 4	(118)
THP-PSMA	GalliProst™	40 μg	1	5	5 min, RT, pH = 6–7	(150)
**Bombesin analogs**							
AMBA	N/A	50 μg	1	1	10 min, 90°C, pH = 3.5–4.0	(151)
DOTA-RM2	N/A	50 μg	1	1–5	10 min, 90°C, pH = 3	(114)
		50 μg	1	1.1	10 min, 100°C, pH = 3	(125)
HBED-CC-PEG_2_-RM26	N/A	40 μg	1	2	5 min, 80°C, pH = 3	(81)
NODAGA-PEG_2_-RM26	N/A	40 μg	1	2	5 min, 80°C, pH = 3	(81)
NeoBOMB1	NeoB	50 ± 5 μg	2	5	7–10 min, 95°C, pH = 3.6–4.0	(122)
**Other peptides/biomolecules**							
NOTA-RGD	N/A	60 μg	1	1	10–15 min, 90°C, pH = 4.0–4.5	(152)
NOTA-Ubiquicidin	N/A	30 nmol	1	2.5	15 min, 90°C, pH = 4	(153)
NOTA-SdAb	N/A	100 μg	1	1–1.1	10 min, RT, pH = 5	(154)
NOTA-hexavalent lactoside	N/A	40 μg	1	0.7–1.5	15 min, RT, pH = 4–5	(155)
**Small molecules**							
BAPEN	N/A	0.25 mg	1	1	10 min, RT, pH = 5.5	(156)
HBED-CC-DiAsp	N/A	10 μg	1	4	10, min, RT, pH = 4.3	(157)
**Biphosphonates**							
EDTMP	Multibone®	25 mg	1	5	30 min, RT	(158)
DOTMP	N/A	400 μg	1	0.5	7 min, 100°C, pH = 4.5	(159)
THP-Pam	N/A	5 μg	1	0.25	5 min, RT, pH = 7	(160)
P15-041	N/A	30 μg	1	4	5 min, RT, pH= 4.5–5.5	(161)
**Particulates**							
MAA/HSA	4 different commercial kits	1–5 mg	1	0.1	15 min, 74 ± 1°C, pH = 4.7	(162)
	Pulmolite®	10 mg	1	5	15 min, 75°C, pH = 5–6	(163)
	HSA Microsphere B20	2.5 mg	1	1.5	20 min, 75°C, pH = 4	(164)
	MAASol®	1.75 mg	1	<1	10 min, 90°C, pH = 4.5	(132)
				1.5	20 min, 75°C, pH = 4	(164)
				<1	10 min, 90°C, pH = 4.5	(132)
	DraxImage® MAA	2.5 mg	1	1	15 min, 75°C, pH = 5.2	(165)
	TCK-PARS-1800	3 mg	1	1.5	8 min, 75°C, pH = 3.9–4.2	(166)
	LyoMAA®	2 mg	1	<1	10 min, 90°C, pH = 4.5	(132)
NanoHSA	NanoAlbumon®	0.5 mg	1	8	20 min, 40°C, pH = 4–4.5	(167)
Phytate	Phytacis®	20 mg	3	1	30 mi, 100°C, pH = 1–2	(168)
SBMP	N/A	20 mg	1	4	10 min, RT, pH = 4.1	(169)

#### Neuroendocrine Tumor Imaging

Somatostatin receptors (SSTRs) are widely expressed in the whole body, but their expression is significantly enhanced in many solid tumors, especially gastro-entero-pancreatic neuroendocrine tumors (GEP-NETs). Thus, radiolabeled somatostatin analogs have been developed to visualize the distribution of receptor overexpression in tumors and/or from a therapeutic perspective (84). Among them, [^68^Ga]Ga-DOTATOC ([^68^Ga-DOTA-Tyr^3^]octreotide), [^68^Ga]Ga-DOTATATE ([^68^Ga-DOTATyr^3^]octreotate), and [^68^Ga]Ga-DOTANOC ([^68^Ga-DOTA^1^NaI^3^]octreotide) have shown themselves particularly useful for neuroendocrine tumor imaging, leading to the approval of the first two for GEP-NET imaging (170). Evaluation of a cold kit-based preparation of ^68^Ga-labeled somatostatin analogs was first described by Mukherjee et al. (171). They consisted of single vial kits containing 50 μg of a lyophilized peptide plus sodium acetate in order to achieve a pH in the range of 3.5–4 upon addition of the ^68^Ga eluate (1 mL of 0.1 M HCl). A small study on 10 patients with a freeze-dried kit-prepared [^68^Ga]Ga-DOTATOC allowed to identify sites of primary and metastatic diseases with good accuracy and specificity (172). Other teams around the world also successfully developed kit formulations for DOTA-somatostatin analogs (113, 173). Positive clinical results with these radiotracers eventually led to the approval of [^68^Ga]Ga-DOTATATE (NETSPOT™) in the United States and [^68^Ga]Ga-DOTATOC (Somakit TOC®) in Europe (174). First clinical investigations with this [^68^Ga]Ga-DOTATOC kit successfully validated its safety and clinical utility (106).

Radiolabeling with DOTA peptides requires heating at over 90°C, and might not represent the best candidate for kit-based radiopharmacy. To achieve rapid labeling under mild conditions, somatostatin analogs based on other more “user-friendly” chelators have been proposed. THP-Tyr^3^-octreotate (THP-TATE) has been labeled with ^68^Ga in <2 min at room temperature, in ≥ 95% radiochemical yield (99). No freeze-dried kit formulation has, however, been reported to date. One of the reasons may be the higher retention in non-target organs and lower tumor-to-liver ratio than for [^68^Ga]Ga-DOTATATE, which limits its interest in clinic. [^68^Ga]Ga-DATATOC, based on a DATA chelator, has been radiolabeled with ^68^Ga in 1–10 min, depending on the post-processing method used, at ambient temperature using a single vial kit containing 13 nm of a peptide derivative (146). In a first-in-human study, it demonstrated higher tumor-to-liver contrast in a NET-patient compared to [^68^Ga]Ga-DOTATOC, while in a subsequent study with 50 patients, it displayed comparable results with [^68^Ga]Ga-DOTANOC (175, 176). It has been hypothesized that somatostatin, antagonists, and other peptides might be more useful than agonists. Actually, after binding to its receptor, an agonist analog is internalized into a cell as a ligand-receptor complex. This internalization allows it to accumulate into the cell. This does only slightly occur for antagonists, and they do not activate the receptor. Antagonists do, however, accumulate more on the target, as a consequence of a greater number of target binding sites for antagonists and a more slowly dissociation kinetics than for agonists, and probably due to a possible ligand rebinding mechanism (36, 84, 177). In that context, ^68^Ga-OPS202 ([^68^Ga]Ga-NODAGA-JR11) has attracted particular attention and demonstrated superiority over [^68^Ga]Ga-DOTATOC (178). Satpati et al. recently reported the development of a cold kit for ^68^Ga-OPS202, formulated with either HEPES or sodium acetate buffer (147). To our knowledge, ^68^Ga-OPS202 prepared this way has not yet been evaluated in humans.

#### Prostate Cancer Imaging

Prostate-specific membrane antigen (PSMA), a type II transmembrane protein on the surface of cancer cells, is considered to be the most interesting antigen in prostate cancer, since it is overexpressed in high-grade tumors, metastases, and hormone-resistant tumors with low concomitant expression (100 to 1,000 times less) in normal tissues (179). PSMA is, therefore, an ideal target for molecular imaging of prostate cancer, especially for the development of small radiolabeled molecules, having fast plasma clearance and generating little background noise (180). Identification of the binding site of PSMA substrates promoted the development of small molecule ligands or inhibitors of PSMA. PSMA inhibitors can be grouped into three families: phosphorus inhibitors, sulfur inhibitors, and urea-based inhibitors. The latter have great affinity and good specificity for PSMA as well as rapid internalization into tumor cells (181). The small molecule Lys-Urea-Glu (Lys-NH-CO-NH-Glu) composed of two amino acids, lysine and glutamate, united by a urea unit is one of them. This pharmacophore has been conjugated with several chelators for labeling with ^68^Ga (182). Among them, HBED-CC linked to the Lys-Urea-Glu motif through a 6-aminohexanoic (Ahx) spacer afforded particularly positive results (183). Eder et al. were particularly interested in the influence that the proportion of isomers could have on the characteristics of the binding of the radiotracer to its target (184). They showed that specific binding to PSMA and internalization of the radiotracer were comparable, whatever the proportion of isomers present. Successful clinical investigations eventually led to FDA approval for the UCLA Biomedical Cyclotron Facility (Los Angeles, CA, United States) and the UCSF Radiopharmaceutical Facility (San Francisco, CA, United States) (119). This imaging agent, indeed, has had a significant impact on prostate cancer management (185).

Preparation of a freeze-dried cold kit formulation for the preparation of [^68^Ga]Ga-HBED-CC-PSMA-11 was first reported by Ebenhan et al. in 2015 (80). The radiotracer was conveniently prepared in 15 min, at room temperature, from a single-vial kit containing 5 μg of HBED-CC-PSMA-11 precursor. It passed all quality control criteria and, in a 15-patient study, was able to detect primary prostate cancer as well as metastatic lesions. Other kit formulations containing HBED-CC-PSMA-11 have been subsequently set up (118, 148). Two commercial kits are now available for investigational studies, as mentioned above, allowing for fast labeling of ^68^Ga in 5 min at room temperature (148, 149). Both kit formulations demonstrated their clinical applicability and are compatible with all commercial ^68^Ge/^68^Ga generators as well as cyclotron production.

Another PSMA derivative, based on the promising THP chelator [tris(hydroxypyridinone)], has also attracted attention. A single-vial kit formulation has been developed by (Theragnostics, Bracknell, UK) (GalliProst™). It contains 40 μg of THP-Glu-urea-Lys(Ahx), which is a higher amount than in PSMA-11 kits, and complete labeling occurs in 5 min at ambient temperature (150). Positive clinical outcomes, i.e., good detection rates in patients with biochemical recurrence coupled with ease of use, led to an ongoing phase 2 clinical trial (120, 186–188). It has to be noted, however, that detection rates are lower than with other PSMA imaging agents.

Gastrin-releasing peptide receptors (GRPRs) are another potential target for prostate cancer imaging and therapy. Various ^68^Ga-labeled peptides targeting these receptors have been reported in the literature, among which are bombesin derivatives, either agonists (i.e., AMBA) or antagonists (i.e., RM2) (189–191). The GRPR antagonist [^68^Ga]Ga-RM2 (RM2=DOTA-4-amino-1-carboxymethylpiperidine-D-Phe-Gln-Trp-Ala-Val-Gly-His-Sta-Leu-NH_2_) has been particularly attractive for prostate cancer imaging. In small cohorts of patients, it emerged as a possible alternative to [^68^Ga]Ga-PSMA-11 imaging, thanks to its different biological processes (192). Its role is, non-etheless, still a matter of debate (193). In view of the potential interest in this radiotracer, several kit formulations have been reported in the literature. Vats et al. developed three single-vial formulations, each adapted to one single ^68^Ge/^68^Ga generator type (114). *In vitro* studies demonstrated that [^68^Ga]Ga-RM2 prepared this way was suitable for GRPR imaging. Soon after, Chastel et al. developed a similar kit formulation (50 μg of peptide precursor, same reaction conditions) usable with a GalliAd® generator (125). Besides demonstrating high uptake in GRP-R-expressing PC-3 cells, they established the importance of excipients (scavenger, bulking agent) in the formulation of freeze-dried kits, since composition without trehalose did not meet criteria for lyophilization.

#### Lung Perfusion Imaging

Albumin particles were the first reported example of cold kit labeling with ^68^Ga, as far as in the end of the 1980s, for imaging pulmonary perfusion with higher resolution and sensitivity than conventional ^99m^Tc SPECT imaging. Experiments were conducted using commercial [^99m^Tc]Tc-MAA kits (162). Since then, different types of albumin particles have been labeled, either macroaggregated albumin or human serum albumin (HSA), still with various commercial ^99m^Tc-kits, and employing a similar procedure to prepare the kits for the labeling with ^68^Ga. The kits were suspended in 5-ml sterile saline, vigorously shaken, then centrifugated to separate particles from stannous chloride and other components, such as polysorbate or free albumin. After centrifugation, the supernatant was discarded and albumin particles were resuspended in a small volume of sterile water or saline and used for labeling with ^68^Ga (164, 170). Some authors also relyophilized the kits before use (165). It was, however, demonstrated that removal of stannous chloride prior to labeling with ^68^Ga was not necessary. Indeed, absence or presence of SnCl_2_ had no impact on labeling efficiency (132). Unfortunately, despite initial promises and possible lower-dose delivery to critical organs, ^68^Ga-MAA or ^68^Ga-HSA has never broken through (166).

To avoid the use of human blood derivatives, starch-based microparticles (SBMPs) have been proposed as a surrogate to albumin particles for lung imaging and labeled with ^99m^Tc (194). Verger et al. reported the labeling of these SBMPs with ^68^Ga, but only investigated their potential use as a pre-therapeutic tool for radioembolization of liver cancer (169). In a similar manner, phytate particles, using a commercial Phytacis® kit for ^99m^Tc, have been proposed as a possible ^68^Ga perfusion imaging agent (168).

#### Other Applications

As mentioned above, GRPR imaging has found interest in prostate cancer diagnostic and staging. GRP receptors are, however, expressed in several other cancers, which could benefit from the use of GRPR-targeting ^68^Ga radiotracers (195). Gastrin-releasing peptide receptors are notably overexpressed in breast and lung cancers. Besides [^68^Ga]Ga-RM2, several other cold kit formulations have been reported with GRPR-addressed peptides. Pandey et al. described a kit formulation with GRPR agonist [^68^Ga]Ga-AMBA ([^68^Ga]Ga-DO3A-CH_2_CO-G-[4-aminobenzoyl]-QWAVGHLM-NH_2_), while Satpati et al. reported the labeling of the antagonist RM26 (D-Phe-Gln-Trp-Ala-Val-Gly-His-Sta-Leu-NH_2_), conjugated either with a NODAGA or an HBED-CC chelator (81, 151). Another promising bombesin derivative is the antagonist NeoBomb1. It is currently the object of a clinical study on patients with oligometastatic gastrointestinal stromal tumors, and is prepared using a kit procedure (196). This cold kit, designed for use with a Galliapharm® generator, enables fast radiolabeling of peptides in <10 min with a 2-vial formulation (122).

Several other kit-based ^68^Ga radiosyntheses have been reported with potentially interesting peptides. Integrin receptor targeting [^68^Ga]Ga-NOTA-RGD, for angiogenesis imaging, has been described by Ebenhan et al. using a single-vial kit formulation (152). Ubiquicidin (UBI 29-41 = TGRAKRRMQYNRR) is an antimicrobial peptide designed as an infection-specific imaging agent when radiolabeled. It was first labeled with ^99m^Tc (197). A ^68^Ga-PET surrogate has subsequently been developed and investigated using a kit-based preparation (153). This small study on 10 patients gave mixed results, as 4 patients gave false negative results possibly because of antibiotic treatment (2 patients) or because of very low microbial population. Studies with a larger number of patients are necessary to assess its usefulness.

Biphosphonates are another important class of imaging agents. They are extensively used for bone imaging, in particular for visualization of skeletal metastases. Several bisphosphonate derivatives have been labeled with ^68^Ga, for which kit formulations have been reported. EDTMP (ethylenediamine tetramethylene phosphonic acid) has been radiolabeled with ^68^Ga using a commercial Multibone® kit, initially designed for use with ^99m^Tc, but its utility remains uncertain (158). ^68^Ga-labeled BPAMD (4-{[bis-(phosphonomethyl))carbamoyl]methyl}-7,10-bis(carboxy methyl)-1,4,7,10-tetraazacyclododec-1-yl)acetic acid) has shown favorable preclinical and clinical properties. The radiotracer, prepared with a kit-based procedure, gave satisfactory results in terms of ease of use and clinical outcome in a small cohort of patients (198). [^68^Ga]Ga-THP-Pam, based on pamidronic acid conjugated to a THP chelator, was easily obtained under mild conditions (5 min, RT, and pH 7) and demonstrated high *in vivo* affinity for bone tissue, comparable to [^18^F]NaF (160). Latest example to date is [^68^Ga]Ga-P15-041, an HBED-CC-derived bisphosphonate (161).

#### Future Developments

Several other ^68^Ga imaging agents demonstrated promising preclinical and clinical outcomes. They are currently prepared manually or through automated procedures, which limits their dissemination. The set-up of cold kit formulations for these radiotracers would, thus, be of great benefit for the medical community. Among the radiotracers of interest, one can cite the peptides neurotensin and exendin-4, for which receptors are overexpressed in several tumor types (199–202). These two peptide derivatives could be of interest particularly in pancreatic cancers. Preliminary clinical results demonstrated the safety and tolerance of a ^68^Ga-labeled agonist neurotensin peptide ([^68^Ga]Ga-NT-20.3) conjugated to a DOTA chelator (203). Antagonist derivatives have also been investigated with various common ^68^Ga chelators (98). It was shown that the DOTAGA chelator gave the best results *in vitro* and *in vivo*, and that the THP chelator, *a priori* the best suited chelator among the ones investigated for kit formulation thanks to its radiolabeling characteristics, led to poor tumor uptake. Several chelators have been conjugated with exendin-4 (DOTA, NODAGA, DFO, and HBED-CC), and some of them are suitable for cold kit preparation (130, 204, 205).

Nevertheless, radiotracers with highest promises are probably the CXCR4 and FAP inhibitor ligands, targeting a tumor microenvironment. Chemokine receptor 4 (CXCR4) is overexpressed in numerous tumor types, and plays a critical role in tumor growth and invasiveness, as well as metastasis (206). The ^68^Ga-labeled CXCR4 antagonist ([^68^Ga]-Pentixafor), prepared automatically through a commercial single-use cassette (207), has revealed itself as a useful clinical tool in hemotologic diseases and in solid tumors, as well as under benign conditions (208–210). Fibrobast activation proteins (FAPs), overexpressed in a variety of tumors, are involved in several tumor-promoting activities and represent a prospective theranostic target. A family of inhibitors of this protein based on a quinolone scaffold has been developed and labeled with several diagnostic and therapeutic radionuclides, such as ^68^Ga (211, 212). Among the FAPI derivatives developed, [^68^Ga]Ga-FAPI-4 and [^68^Ga]Ga-FAPI-46, with a DOTA chelator, led to impressive results (213–215). New derivatives suitable for cold kit radiolabeling were recently described, one with a NOTA chelator (FAPI-74) (216), and another with the DATA^5M^ chelator (217).

## Conclusion

Radiolabeling of clinically useful ^68^Ga-PET imaging agents with ready-to-use lyophilized cold kits may contribute to their worldwide dissemination, in particular in small or low-budget centers not equipped with an elaborate and expensive set-up, enabling for earlier and better diagnostics and treatment response assessment in the context of personalized medicine. Kit-based synthesis has proven to be “user-friendly,” trustworthy, robust, and cost-effective. New chelators such as THP, DATA, and NOTA derivatives, enabling “instant kit” procedures, are particularly appealing in that regard. The repertoire of applications is steadily increasing, following clinical demand. Cold kit formulations of most promising new radiotracers such as CXCR4 and FAPI ligands are eagerly awaited.

In large centers with high patient throughput, however, automated modules may most probably remain the favored production mode for ^68^Ga-labeled imaging agents, maybe in conjunction with kits, based on better GMP-compliance, and for radioprotection reasons. Another reason might be early access to newly designed radiotracers, since kit formulations will only be developed for already established tracers, having demonstrated their clinical effectiveness.

Eventually, effective development of ^68^Ga cold kits will rely on easy access to ^68^Ga, either through a generator or a cyclotron, simplicity, and robustness of ^68^Ga incorporation into novel radiopharmaceuticals, successful clinical applications, and strong medical industry support.

## Author Contributions

The author confirms being the sole contributor of this work and has approved it for publication.

## Funding

This study was partly supported by Labex IRON (Grant No: ANR-11-LABX-0018) and Rennes Métropole (Grant No: 09 EML 2014).

## Conflict of Interest

The author declares that the research was conducted in the absence of any commercial or financial relationships that could be construed as a potential conflict of interest.

## Publisher's Note

All claims expressed in this article are solely those of the authors and do not necessarily represent those of their affiliated organizations, or those of the publisher, the editors and the reviewers. Any product that may be evaluated in this article, or claim that may be made by its manufacturer, is not guaranteed or endorsed by the publisher.
